# Rapid PCR Method for the Selection of 1,3-Pentadiene Non-Producing *Debaryomyces hansenii* Yeast Strains

**DOI:** 10.3390/foods9020162

**Published:** 2020-02-07

**Authors:** Eva-María Rivas, Petra Wrent, María-Isabel de Silóniz

**Affiliations:** 1 Department of Genetics, Physiology and Microbiology. Biological Sciences Faculty. Complutense University of Madrid. José Antonio Nováis, 12. 28040 Madrid, Spain; evarifer@gmail.com (E.-M.R.); pwrent@ucm.es (P.W.); 2 CEI Campus Moncloa, UCM-UPM, 28040 Madrid, Spain

**Keywords:** strains-selection, 1,3-pentadiene, sorbate, spoilage-yeast, food-preservation

## Abstract

To prevent microbial growth and its consequences, preservatives such as sorbic acid or its salts, commonly known as sorbates, are added to foods. However, some moulds and yeasts are capable of decarboxylating sorbates and producing 1,3-pentadiene. This is a volatile compound with an unpleasant “petroleum-like “odour, which causes consumer rejection of the contaminated products. In this work, we studied the production of 1,3-pentadiene in 91 strains of the yeast *Debaryomyces hansenii*, and we found that nearly 96% were able to produce this compound. The sequence of the *FDC1Dh* gene was analysed showing differences between 1,3-pentadiene producer (P) and non-producer (NP) strains. A specific PCR assay with degenerated primers based on the gene sequence was developed to discern NP and P strains. It was tested on *D. hansenii* strains and on some physiologically related species frequently isolated from foods, such as *D. fabrii*, *D. subglobosus* and *Meyerozyma guillermondii*. This method could be applied for the selection of NP *D. hansenii* strains, useful in biotechnological food production and as a biocontrol agent.

## 1. Introduction

Yeasts are beneficial organisms that contribute to the production of certain foods and beverages [1,2,3,4] but can cause spoilage [5,6]. There is an increased concern about the spoilage produced by yeasts [7]. They are able to grow in products with low water activity, pH, and low temperatures [7]. Moreover, few species are able to grow in the presence of preservatives such as low molecular weight weak acids [5,8]. 

Sorbic acids and their salts are weak acid preservatives whose fungistatic activities are favoured at low pH, where they are found in their undissociated forms. The FDA (U.S. Food and Drug Administration), JEDFA (Joint FAO/WHO Expert Committee on Food Additives) and SCF (Scientific Committee in Food) evaluations consider these preservatives to be among the safest and, according to the EU EFSA Panel, the most effective. However, the microbial decarboxylation of sorbates in a single step produces volatile 1,3-pentadiene that has a petroleum-hydrocarbon-like unpleasant off-odour. Fungal sorbate degradation was first demonstrated on *Penicillium* strains isolated from cheddar cheese, all the strains isolated were able to eliminate the sorbic acid [9]. Later in the 1990s, more yeast strains were described as 1,3-pentadiene producers, including *D. hansenii* strains, which were isolated from cheese, margarine, butter or marzipan [10,11,12,13]. *D. hansenii* appears in the inventory of microorganisms with technological benefits for its use in food fermentation [1,2,3,14]. It is also used in cured meat, where it has been proposed as a starter [15,16] and as a biocontrol agent [17,18,19,20]. *D. hansenii’s* effectiveness as a biocontrol agent is well studied but its ability to degrade sorbates if strains survey and remain in the final product has not been analysed. Therefore, a method that distinguishes between 1,3-pentadiene producer (P) or non-producer strains (NP) could be of great interest to the industry.

Detection of 1,3-pentadiene is feasible by sensorial, gas chromatography coupled to mass spectrometry (GC-MS) or MWIR (Mid-Wave IR) devices [11,21,22,23,24]. These techniques and their implementation are time-consuming and expensive for 1,3-pentadiene detection. 

The decarboxylation of sorbic acid in 1,3-pentadiene requires the removal of the carboxyl group of the molecule. The molecular basis of 1,3-pentadiene production has been studied mainly in strains of *Aspegillus niger* and *Saccharomyces cerevisiae* [25,26] and it was shown that it requires the activity of a Pad1 enzyme (named in this work as Phenylacrylic Acid Decarboxylase). Goodney y Tubb [27] described that the *PAD1* gene (named in this work as *POF1*, Fenolic Off Flavour) encoded for a ferulic acid decarboxylase. Sorbic acid is not considered a phenylacrylic acid as ferulic, cumaric or cinnamic acids but shares some structural characteristics with them, such as a carboxylic group and an aliphatic chain with two double bonds. Further studies in *Aspergillus* reported that a second gene was involved [28]. It is an oxidative decarboxylation produced by two enzyme systems: PAD1 and FDC1 (Ferulic Acid Decarboxylase) [29]. More recently, a positive relation has been reported between the number of single nucleotide polymorphisms of *PAD1* and *FDC1* and ferulic acid decarboxylation in several industrial yeast strains [30]. The aim of this work was to develop a simple method for *D. hansenii* NP strains selection using a new PCR protocol based on the *FDC1Dh* gene.

## 2. Materials and Methods

### 2.1. Yeast Strains and Culture Conditions

A total of 129 strains, some of them from 1,3-pentadiene spoiled foods, were used in this work from different Culture Collections or isolated in our laboratory (see Supplementary Material, Table S1). Strains were cultured at 28 °C in Yeast Morphology Broth (YMB) and routinely maintained on the same culture medium plus Agar (YMA): 0.5% (w/v) yeast extract (Difco Laboratories, Detroit, MI, USA), 0.3% (w/v) proteose-peptone No.3 (Difco), 0.3% (w/v) malt extract (Difco), 1% (w/v) glucose (Panreac Quimica S.A., Barcelona, Spain), and 2% (w/v) agar. 

For 1,3-pentadiene detection, bottles (20 mL chromatographic magnetic screw-capped, LLG Labware, Meckenheim, Germany) containing 9 mL of YMB pH 7 supplemented with potassium sorbate 0.75g/L (Scharlau, Barcelona, Spain) [24] were inoculated with 1 mL of a saline solution suspension of the yeasts (ca 6 McFarland). The bottles were incubated at 28 °C for 4 days.

### 2.2. 1,3-Pentadiene Detection 

Two methods were used for 1,3-pentadiene detection. (1) GC-MS (GC:Varian CP-3800) coupled with Mass Spectrometry (MS:Saturno 2200 GC/MS/MS in automatic mode and with an automatic CombiPal Splitless injector: Two hundred microliters of headspace volatile compounds were analysed. Pure 1,3-pentadiene was used as an internal standard (50% mixture cis-trans isomers, Aldrich-Chemical, Wisconsin, USA). (2) A sensory method: Three independent experts introduced a needle into the headspace of each culture and sniffed the sample to detect the “petroleum smell” as previously described [24]. Once the accuracy of the sensory method was verified, it was applied to the rest of the strains listed in Table 1.

### 2.3. Primer Design and Sequencing

For the primer design, we used the putative homologous *FDC1 Saccharomyces cerevisiae* region (1500 bp) [29,30] present in *D. hansenii* as a target, whose sequence was obtained from NCBI GenBank accession No. XM_461563.1 [31]. Based on the nucleotide sequence found in both species, the primers FDC1_Dh_Full_Fw 5′ CTATTTATATCCGTACGCAGACC 3′ and FDC1_Dh_Full_Rv 5′ TAATATGAGCAATTTAAGACCAGAG 3′ were designed. With the objective of analysing differences in sequence between the 1,3-pentadiene *D. hansenii* producing (P) or non-producing strains (NP), a DNA template was obtained as described by Lõoke et al. [32]. PCR amplifications were performed in an Eppendorf Mastercycler Gradient (Eppendorf, Hamburg, Germany) following the protocol described below. After purification (Ultraclean™ PCR clean-up Kit (MO-BIO, Larsband, USA), 80 µL of all positive amplicons were sequenced (ABI PRISM 3730XL DNA Analyzer (Applied Biosystem, Foster, CA, USA). All sequences were aligned with ApE (A plasmid Editor, M.W. Davis) which is freely available [33]. After detecting the differences in the sequence between (P) and (NP), a degenerate primer FDC1_Dh_Pentadien 5′CGTAGACCYTTCTCATAATAGCA 3′, where Y = C or T was designed to amplify a 130 bp intermediate region which was used together with the reverse primer FDC1_Dh_Full_Rv in the PCR reaction described below. The primers used were prepared by Conda Labs-Spain Portal at Integrated DNA Technologies. For validation purposes, each strain was tested at least twice.

### 2.4. PCR Conditions

DNA amplifications were carried out in 25 µL reactions containing 50–100 ng genomic DNA, 1.25 μL of each primer (20 µM), 12.5 µL NZYtaq2x colourless Mastermix (NzyTech, Lisbon, Portugal) and nuclease-free water to a final volume of 25 µL. Different annealing temperatures were tested, ranging from 52 °C to 68 °C. PCR conditions were as follows: initial denaturation at 94 °C for 5 min; 30 cycles of 94 °C for 1 min, 45 sec at the T_m_ selected, 72 °C for 45 sec; and then 1 cycle of 72 °C for 8 min. PCR-amplified DNA fragments were separated in 1% (w/v) agarose gels (Bio-Rad) and visualised under UV light. The GeneRuler 100bp plus DNA Ladder (MBI Fermentas) was used as a molecular size marker. 

### 2.5. Analysis of Protein Sequences

The sequences of the *FDC1Dh* of *D. hansenii* were converted into their corresponding amino acid sequence with the ApE programme, taking into account that the CUG codon of *D. hansenii* codes for serine instead of leucine. Subsequently, these proteins were aligned using MegAlign—CLUSTAL method, (Lasergene, Madison, WI, USA) and web Clustal Omega [34] and Esprit 3.x [35] web applications. 

## 3. Results

The ability to produce 1,3-pentadiene, indicating sorbate degradation, was studied in selected species (Table 1). The results obtained by gas chromatography coupled with mass spectrometry (GC-MS) were compared to a sensorial method [24] based on Casas et al. [36]. In the chromatographic analysis, the same peak was obtained both in the gas collected in the free headspace of the cultures and in the control samples containing 1,3-pentadiene. The fragmentation of the mass spectrum of that peak presents characteristic ions of 39, 53 and 67 m/z (see Supplementary Material, Figure S1). As can be seen, both GC-MS and olfactory sensorial methods provided the same results (Table 1). In the remaining strains, 1,3-pentadiene was detected using the olfactory sensorial method as described in the Material and Methods section (Table 2). We found only four out of 91 *D. hansenii* strains that did not produce 1,3-pentadiene (NP, non-producers), and therefore nearly 96% of the strains of this yeast were able to produce this volatile compound.

Next, to achieve our goal of obtaining specific primers for the detection of *D. hansenii* strains producing 1,3-pentadiene, we designed a primer pair based on a *S. cerevisiae FCD1* gene sequence to amplify putative homologous gene from *D. hansenii* gDNA [29,30]. The amplified region presents a sequence identity of 66% with the *FDC1* gene of *S. cerevisiae*. The best result for the amplification of the *FDCDh* region was obtained after 30 cycles and with an annealing temperature of 59 °C. A single fragment of about 1542 bp was amplified from all of the *D. hansenii* strains. By analysing these sequences we observed nucleotide polymorphism of the *FDC1Dh* gene between 1,3-pentadiene producer (P) and non-producer (NP) strains (see Supplementary Material, Figure S2). Many nucleotide differences were related to amino acid changes (Table 3). Additionally, and most importantly, all NP strains contain at least one deletion in the nucleotide sequence of the *FDC1Dh* gene (Table 3). Specifically, the deletion of adenine or guanine in position 383 alters the reading frame and consequently, it would be responsible for a premature STOP codon. Only one NP strain, PR5, had two more deletions in the positions 281 and 1234, the first of them being responsible for an alteration of the reading frame and a premature STOP (see asterisks in Figure S2).

As mentioned, one of the objectives of this work was to develop a simple method for differentiating P and NP strains of *D. hansenii* by PCR. For this, an FDC1_Dh_Pentadien degenerated primer was designed, based on the sequences of the *FDC1Dh* gene of the strains, as described in the Material and Methods section. It comprises the position 127 where a base change was detected in P and NP strains. The primer also contains a Y in position 125 that hybridises with C or T present in the sequence of P or NP strains, respectively (see Supplementary Material, Figure S2).

**Figure 1:**
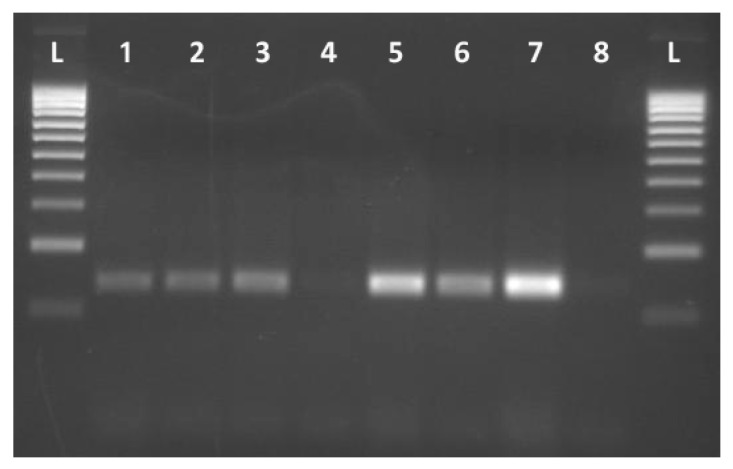
PCR amplification result obtained with primer pair Figure 1. Dh_Full_Rv. L: 100bp ladder. Lines 1-3, 5-7: *Debaryomyces hansenii* 1,3-pentadiene producer strains: CECT 11369T, Es4, EPEC 1.3, CECT 10352, CECT 10378, CH2, respectively. Line 4: *D.hansenii* 1,3-pentadiene no producer strain: J-12; Lane 8: Negative control.

The specificity of the primers and the PCR protocol developed was tested on DNA templates obtained from yeast strains listed in Supplementary Material, Table S1. All P strains of *D. hansenii* gave a positive result with clear amplicons of 130 bp, whereas no amplification was found in NP strains (Figure 1). The rest of the yeast species included in the study showed no amplification, although they were 1,3-pentadiene producing strains (Table 1), supporting the specificity of developed PCR assay for *D. hansenii* strains.

## 4. Discussion 

*Debaryomyces hansenii* shows a dual role in the food industry. It has different biotechnological applications, but it is also capable of spoiling certain products. Among the positive aspects, the yeast is considered a promising alternative to chemical fungicides used in agriculture and several strains have been proposed as biological control agents [17,18,19,20]. However, if potentially spoiling strains are used, such as those that degrade sorbates, the yeasts present on the fruits or vegetables could remain in the final products obtained [37,38,39]. Taking into account that the decarboxylation of sorbic acid is not a property of the yeast species but of the strain [40], the selection of strains that do not produce unpleasant “petroleum-like“off-odours would be of importance for quality and safety reasons. According to the International Chemical Safety Card (CAS No. 504-60-9), a low exposure (concentrations) of 1,3-pentadiene does not have an adverse effect on humans. Nevertheless, the problem is not only the production of a compound with an offensive odour but the fact that the antimicrobial action of the sorbates disappears and other undesirable fungi or bacteria can grow on the food.

Table 2 shows that the production of volatile 1,3-pentadiene is a common feature in the *D. hansenii* strains studied. Under the study conditions, a conversion of 45% of the sorbate into 1,3-pentadiene was measured by chromatography in *D. hansenii* (data not shown). The ability to produce 1,3-pentadiene is a strain characteristic, surprisingly nearly 96% of 91 *D. hansenii* strains analysed were able to produce this compound. Thus, many strains of *D. hansenii* can cause spoilage. Given that both methods, chromatographic and sensorial, need isolation and cultivation as well as another subsequent cultivation for four days with sorbates, a search for the differences between strains was conducted to develop a fast and accurate molecular method. Based on the *PAD*1 sequence, we previously developed a molecular method for the rapid detection of *D. hansenii* species [41]. However, when beginning this work we did not find differences in the *PAD*1 sequence between (P) and (NP) strains (data not shown). We thus focused our study on the *FDC1* gene. In this work, we describe for the first time a *D. hansenii* putative homologue sequence of the *S. cerevisiae FCD1/YDR539W* gene [42] related to the decarboxylation of sorbates. We developed a PCR protocol based on the differences in the *FDC*1 sequence between (P) and (NP) strains. The primers FDC1_Dh_Pentadien and FDC1_Dh_Full_Rv developed in this assay produce a clear single fragment of 130 bp in all (P) *D. hansenii* strains tested (Table 2), and no false negatives were detected. Additionally, no false positives were found in the other 21 species included in the study. For the industry and control laboratories, this method is easier, quicker and less tedious than the sensorial method, as well as less expensive than the chromatographic method. A 24 h culture, instead of four days, is the time required by the PCR method to differentiate between *D. hansenii* 1,3-pentadiene producer strains and non-producer strains.

## Figures and Tables

**Table 1:** 1,3-pentadiene detection in strains of selected species using the sensory method [24] and chromatography (GC/MS) assays.

Species	Strains	Sensorial Detection	GC/MS
*D. hansenii*	CECT 11369^T^	+	+
	Es 4	+	+
	J-12	-	-
	PR 5	-	-
*D. fabryi*	CECT 11370^T^	+	+
	PR 66	+	+
*Z. rouxii*	T2R	+	+
	Bch	+	+
	TYN 1.3	-	-
	CYC 1484	-	-
*S. cerevisiae*	BY 4747	+	+
	Y05833 (ΔPAD1)	-	-
	ATCC 7754	+	+
	EPO 1.1.2	-	-

ATCC: American Type Culture Collection; CECT: Colección Española de Cultivos; T: Type strain.

**Table 2:** Sensorial results for 1,3-pentadiene producing and non-producing yeast species and strains. PCR amplification with specific primers for the differentiation between 1,3-pentadiene producers and non- producers.

Species/ Strains	1,3-Pentadiene Production	Amplification with Primers FDC1_Dh_Pentadien and FDC1_Dh_Full_Rv
***Debaryomyces hansenii***		
CECT11369^T^, CECT10026, CECT10352, CECT10378, CBS1102, CYC1265, CYC1307, Es 4, J-01, J-09, J-11, J-15, J-16, J-17, CH2, Pr11, Pr13, EPEC1.3, EPEC4, E.2, 29C1.2, 29Inf1, V1.1, V1.2, V1.3, V1.4, V1.6, V1.7, V1.8, V1.9, V1.10, V2.2, V2.4, V.2.5, V2.6, V2.7, V2.8, V2.10, V3.1, V3.3, V3.4, V3.5, V3.6, V3.7, V3.8, V3.9, V3.10, A4.1, A4.2, A5.1, A5.2, A8.1, A8.2, ent 1, ent 2, ent 9, ent 50.3,ent 56, ent 64.1, ent 64.5, ent 64.6, ent 81.1, ent 81.2, ent 15, ent 19, ent 24, ent 55, ent 63, ent 65, Rec1.1, Rec1.3, Rec2.3, Rec2.4, Rec9.1, Rec9.2, Rec11.5, Rec13.1, Rec13.3, ent 23, ent 25, ent 28, ent 95.1, ent 96.1, ent 102.1, ent 102.2, ent 102.4, ent 102.5	+	+
***Debaryomyces hansenii***	_	_
CECT10517, CBS1792, J-12, Pr5		
**Other yeast species**		
***Debaryomyces fabryi*** CECT11370^T^, CECT11365, CBS 6066. ***Debaryomyces subglobosus*** CBS1796^T^, CBS792	+	_
***Saccharomyces cerevisiae*** ATCC7754, YAA1, ***Wickerhamomyces anomalus*** CECT1114^T^, CECT10320		
***Zygosaccharomyces rouxii*** CECT1232^T^, Bch, T2R		
***Hanseniaspora uvarum*** CECT10389, YAb. ***Issachenkia orientalis***, Pim A, PR 3. ***Kregervanrija delftensis*** CECT10238^T.^ ***Lachancea cidri*** CECT10657^T^,. ***Lachancea fermentati*** CECT10382^T^ CECT10678. ***Meyerozyma guilliermondii*** CECT1456^T^. ***Millerozyma farinosa*** CECT1456^T^. ***Ogatea angusta*** CECT10220. ***Priceomyces carsonii*** CECT10227^T^, CECT10230. ***Pichia fermentans*** CECT1455^T^. ***Pichia membranifaciens*** CECT1115^T^. ***Saccharomyces cerevisiae*** CYC1172, CYC1220. ***Schwanniomyces etchelsii*** CECT11412. ***Torulaspora delbrueckii*** CYC1391^T^, CYC1176. ***Wickerhamomyces anomalus*** CECT1112. ***Yarrowia lipolytica*** PR 7, PR 12. ***Zygosaccharomyces bailii*** CECT1898^T^, CECT11042. ***Zygosaccharomyces mellis*** CECT10066.	_	_

ATCC: American Type Culture Collection; CBS: Centraalbureau voor Schimmelcultures; CECT: Colección Española de Cultivos. ^T^: Type strain. +, 1.3-pentadiene production or amplification with primers pair. -, non produces 1,3 pentadiene or non amplify with primers pair.

**Table 3:** Nucleotide polymorphisms in gene *FDC1Dh* that produce amino acid changes in the putative protein sequence. The numbers indicate the nucleotides positions in the gene.

Nucleotide	127	145	156	281	328	362	367	383		458
	G-A	C-T	A-T	*	A-G	T-C	C-A	G-A	*	A-C
***D. hansenii* 1,3-pentadiene producer strains**										
CECT 11369T										
CECT 10352		+						+		
CECT 10386			+		+	+	+			+
CH2			+		+	+	+			+
EPEC 1.3		+						+		
***D. hansenii* 1,3-pentadiene no producer strains**										
CECT 10517	+		+		+	+	+		+	+
CBS 1792	+		+		+	+	+		+	+
J-12	+		+		+	+	+		+	+
PR 5	+		+	+	+	+	+		+	+
Nucleotide	733	775	798	1127	1183	1234	1251	1329	1389	1434
	G-A	A-G	T-A	A-G	C-A	*	T-A	G-A	A-C	A-T
*D. hansenii* 1,3-pentadiene producer strains										
CECT 11369T					+					
CECT 10352	+			+			+	+	+	
CECT 10386		+	+	+			+	+	+	
CH2		+	+		+					
EPEC 1.3	+			+			+	+	+	+
*D. hansenii* 1,3-pentadiene no producer strains										
CECT 10517		+	+	+			+	+	+	+
CBS 1792		+	+	+		+	+	+	+	+
J-12		+	+	+			+	+	+	+
PR 5		+	+	+		+	+	+	+	+

+, Substitution in amino acid; *,Nucleotide deletion.

## Supplementary Materials

The following are available online at https://www.mdpi.com/2304-8158/9/2/162/s1, Figure. S1: Gas chromatogram (above) and mass spectra (below) from the headspace gas of a suspension in YMB of pure 1,3-pentadiene (A) and from the head gas of D. hansenii CECT 11369T cultured on YMB with 0.75 g/l potassium sorbate (B), Figure. S2: Part of the FDC1Dh nucleotide alignment in selected strains of D. hansenii including the most significant base changes. In blue, the producing strains of 1,3-pentadiene and in orange, the non-producing strains. In red, the different bases are highlighted and framed with a black rectangle. The stars show where there is nucleotide deletion. The black numbers indicate the position of each nucleotide within the gene, Table S1: Yeast species and strains used in this study and origin.
