# Supplementary material for: Rapid PCR Method for the Selection of 1,3-Pentadiene Non-Producing Debaryomyces hansenii Yeast Strains
Source: Foods. 2020 Feb 7;9(2):162. doi: 10.3390/foods9020162 (PMC7074485; doi:10.3390/foods9020162)
Supplement: Supplementary file 1 [file foods-09-00162-s001.zip › supplementary/Fig S1 with legend.docx]

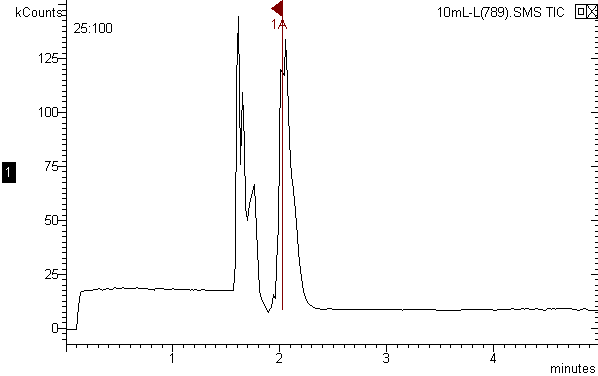

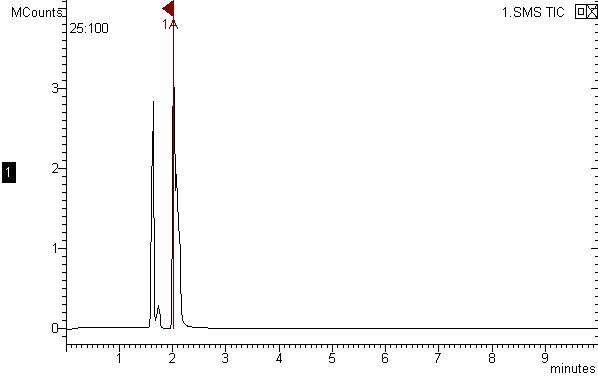

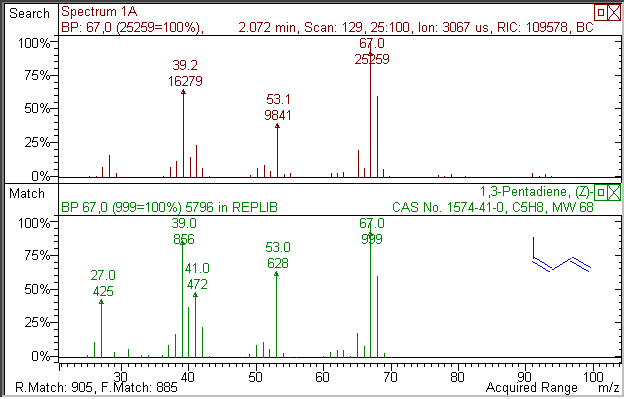

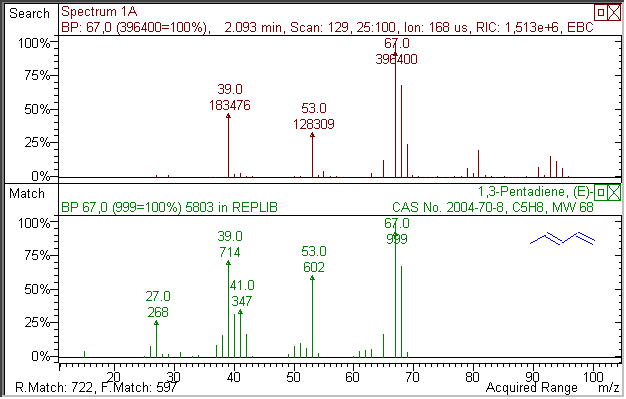


**A**

**B**

Figure S1

**Fig. S1.** Gas chromatogram (above) and mass spectra (below) from the headspace gas of a suspension in YMB of pure 1,3-pentadiene (A) and from the head gas of *D. hansenii* CECT 11369^T^ cultured on YMB with 0.75 g/l potassium sorbate (B).
