# Supplementary material for: Rapid PCR Method for the Selection of 1,3-Pentadiene Non-Producing Debaryomyces hansenii Yeast Strains
Source: Foods. 2020 Feb 7;9(2):162. doi: 10.3390/foods9020162 (PMC7074485; doi:10.3390/foods9020162)
Supplement: Supplementary file 1 [file foods-09-00162-s001.zip › supplementary/Figure S2 with legend.docx]

Figure S2


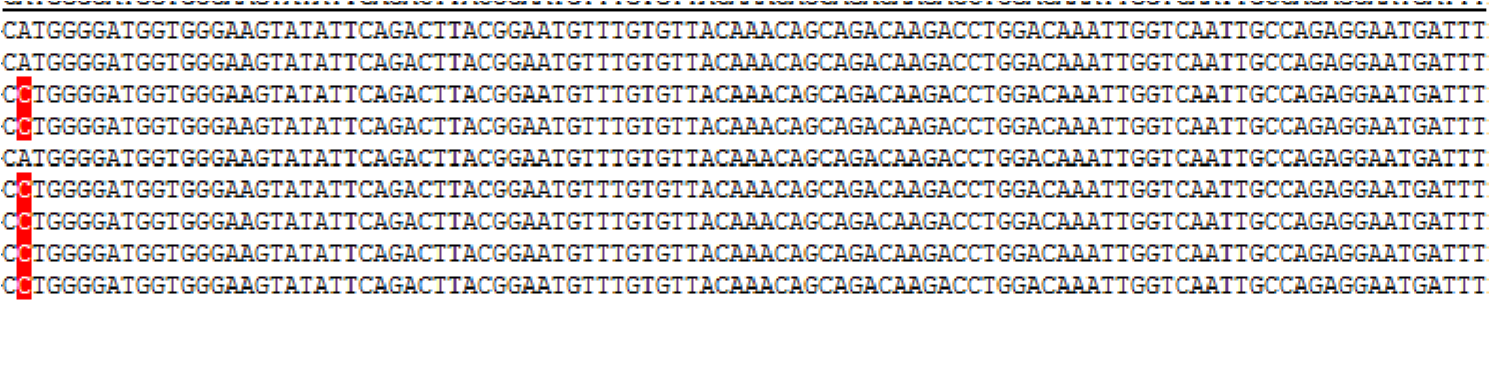


**CECT 11369T**

**CECT 10352**

**CECT 10386**

**CH 2**

**EPEC 1.3**

**CECT 10517**

**CBS 1792**

**J**

**-**

**12**

**PR 5**

457


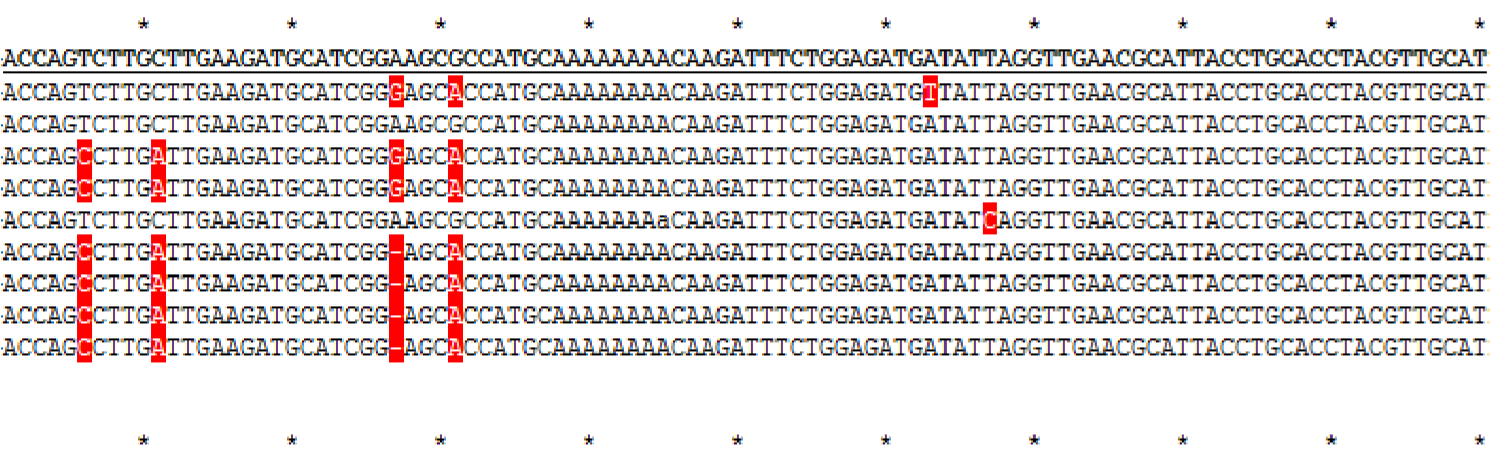


**CECT 11369T**

**CECT 10352**

**CECT 10386**

**CH 2**

**EPEC 1.3**

**CECT 10517**

**CBS 1792**

**J**

**-**

**12**

**PR 5**

357

456

**383**


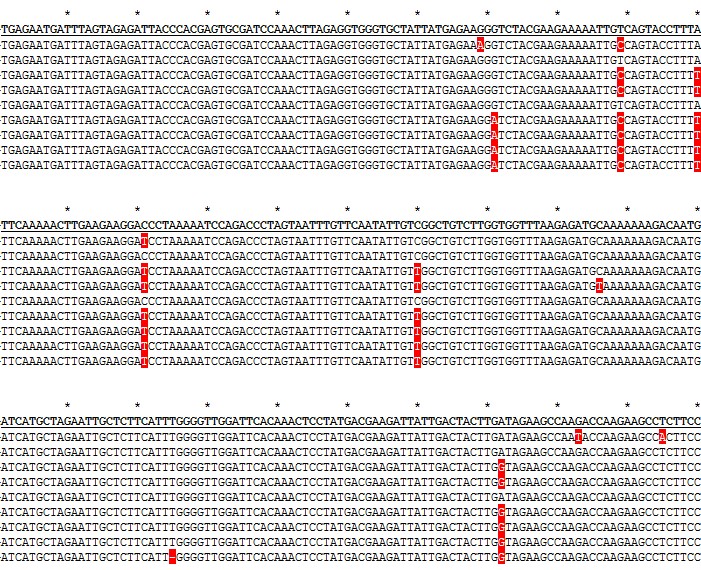


**CECT 11369T**

**CECT 10352**

**CECT 10386**

**CH 2**

**EPEC 1.3**

**CECT 10517**

**CBS 1792**

**J**

**-**

**12**

**PR 5**

**CECT 11369T**

**CECT 10352**

**CECT 10386**

**CH 2**

**EPEC 1.3**

**CECT 10517**

**CBS 1792**

**J**

**-**

**12**

**PR 5**

**CECT 11369T**

**CECT 10352**

**CECT 10386**

**CH 2**

**EPEC 1.3**

**CECT 10517**

**CBS 1792**

**J**

**-**

**12**

**PR 5**

57

156

157

256

257

**127**

**281**


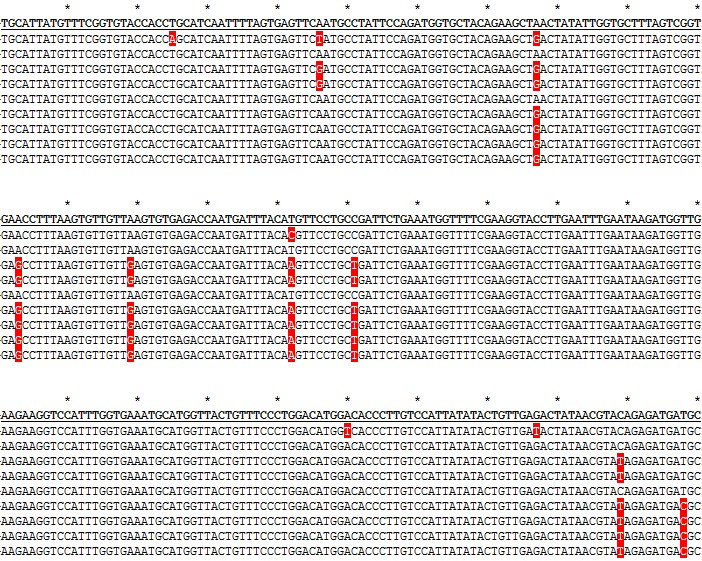


**CECT 11369T**

**CECT 10352**

**CECT 10386**

**CH 2**

**EPEC 1.3**

**CECT 10517**

**CBS 1792**

**J**

**-**

**12**

**PR 5**

**CECT 11369T**

**CECT 10352**

**CECT 10386**

**CH 2**

**EPEC 1.3**

**CECT 10517**

**CBS 1792**

**J**

**-**

**12**

**PR 5**

**CECT 11369T**

**CECT 10352**

**CECT 10386**

**CH 2**

**EPEC 1.3**

**CECT 10517**

**CBS 1792**

**J**

**-**

**12**

**PR 5**

6

57

756

757

856

857

9

56

**954**


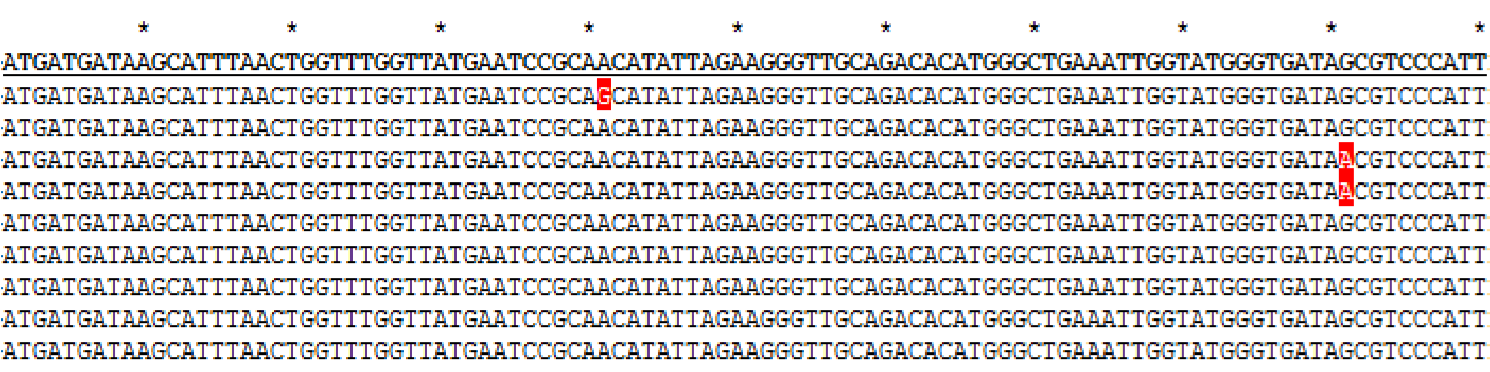


**CECT 11369T**

**CECT 10352**

**CECT 10386**

**CH 2**

**EPEC 1.3**

**CECT 10517**

**CBS 1792**

**J**

**-**

**12**

**PR 5**

557

656


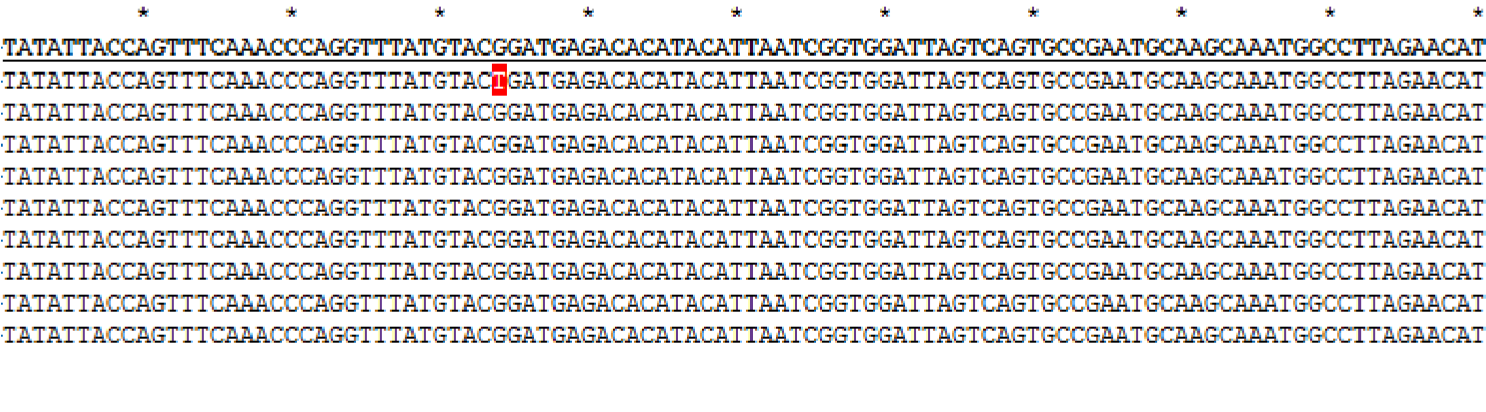


**CECT 11369T**

**CECT 10352**

**CECT 10386**

**CH 2**

**EPEC 1.3**

**CECT 10517**

**CBS 1792**

**J**

**-**

**12**

**PR 5**

957

1056

**Fig. S2.** Part of the *FDC1Dh* nucleotide alignment in selected strains of *D. hansenii* including the most significant base changes. In blue, the producing strains of 1,3-pentadiene and in orange, the non-producing strains. In red, the different bases are highlighted and framed with a black rectangle. The stars show where there is nucleotide deletion. The black numbers indicate the position of each nucleotide within the gene.
