# Supplementary material for: Rapid PCR Method for the Selection of 1,3-Pentadiene Non-Producing Debaryomyces hansenii Yeast Strains
Source: Foods. 2020 Feb 7;9(2):162. doi: 10.3390/foods9020162 (PMC7074485; doi:10.3390/foods9020162)
Supplement: Supplementary file 1 [file foods-09-00162-s001.zip › supplementary/Table S1.docx]

**Table S1.** Yeast species and strains used in this study and origin. .

| **Species** | **Strains** | **Origin** |
| --- | --- | --- |
| ***Debaryomyces hansenii*** | **CECT11369^T^** | **Carlsbergs laboratories** |
|  | CECT10026 | Salt cod |
|  | CECT10352 | Tomato |
|  | CECT10378 | Date |
|  | CECT10517 | Alpechin |
|  | CBS1792 | Chilled beef |
|  | CBS1102 | Beef-and-pork sausage |
|  | CYC1265 | Canned aubergine |
|  | CYC1307 | Unknown |
|  | Es 4 | Marzipan |
|  | J-01, J-09, J-11, J-15, J-16, J-17 | Ham |
|  | J-12 | Ham |
|  | CH2 | Spanish sausage |
|  | Pr5 | Spanish sausage |
|  | Pr11, Pr13 | Meet |
|  | EPEC1.3, EPEC4.E.2, 29C1.2 | Cheese |
|  | 29Inf1  V1.1, V1.2, V1.3, V1.4, V1.6, | Cheese brine |
|  | V1.7, V1.8, V1.9, V1.10, V2.2  V2.4, V.2.5, V2.6, V2.7, V2.8  V2.10, V3.1, V3.3, V3.4, V3.5,  V3.6, V3.7, V3.8, V3.9, V3.10,  A4.1, A4.2, A5.1, A5.2, A8.1, A8.2 ent 1, ent 2, ent 9, ent 50.3 | Spoilage cheese (pigments) |
|  | ent 56, ent 64.1, ent 64.5, ent 64.6, ent 81.1, ent 81.2 ent 15, ent 19, ent 24, ent 55, | Spoilage cheese (gas) |
|  | ent 63, ent 65, Rec1.1, Rec1.3 Rec2.3, Rec2.4, Rec9.1, Rec9.2  Rec11.5, Rec13.1, Rec13.3 ent 23, ent 25, ent 28, ent 95.1 ent 96.1, ent 102.1, ent 102.2 ent 102.4, ent 102.5 | Spoilage cheese (yeast growth) |
| **Other Yeast species** |  |  |
| ***Debaryomyces fabryi*** | CECT11370^T^ | Interdigital mycotic lesion |
|  | CECT11365 | Dry white wine |
|  | CBS 6066 | Tanning fluid |
| ***Debaryomyces subglobosus*** | CBS1796^T^ | Skin lesion |
|  | CBS792 | Infected nail |
| ***Hanseniaspora uvarum*** | CECT10389 | Grape juice |
|  | YAb | Cake |
| ***Issachenkia orientalis*** | Pim A | Paprika powder |
|  | PR 3 | Spanish sausage |
| ***Kregervanrija delftensis*** | CECT10238^T^ | Cider |
| ***Lachancea cidri*** | CECT10657^T^ | Cider |
| ***Lachancea fermentati*** | CECT10382^T^ | Alpechín |
|  | CECT10678 | Drosophila sp |
| ***Meyerozyma guilliermondii*** | CECT1456^T^ | Insect frass |
| ***Millerozyma farinosa*** | CECT1447^T^ | Jopen beer |
| ***Ogatea angusta*** | CECT10220 | Drosophila pseudoobscura |
| ***Priceomyces carsonii*** | CECT10227^T^ | Slime flux of Quercus kelloggii |
|  | CECT10230 | Slimy bottled wine |
| ***Pichia fermentans*** | CECT1455^T^ | Buttermilk |
| ***Pichia membranifaciens*** | CECT1115^T^ | Elm exudate |
| ***Saccharomyces cerevisiae*** | ATCC7754 | Fleischmann baker's yeast |
|  | YAA1 | Yoghurt |
|  | CYC1172 | Orange juice |
|  | CYC1220 | Sediment of wine |
| ***Schwanniomyces etchelsii*** | CECT11412 | Fermenting cucumbers |
| ***Torulaspora delbrueckii*** | CYC1391^T^ | Unknown |
|  | CYC1176 | Naturell yoghurt |
| ***Wickerhamomyces anomalus*** | CECT1114^T^ | Unknown |
|  | CECT1112 | Unknown |
|  | CECT10320 | White wine |
| ***Yarrowia lipolytica*** | PR 7 | Spanish sausage |
|  | PR 12 | Meat |
| ***Zygosaccharomyces bailii*** | CECT1898^T^ | Apple juice |
|  | CECT11042 | Grape must |
| ***Zygosaccharomyces mellis*** | CECT10066 | Honey-bee |
| ***Zygosaccharomyces rouxii*** | CECT1232^T^ | Concentrated must |
|  | Bch | Chocolate cake |
|  | T2R | Fruit nougat |

ATCC: American Type Culture Collection; CBS: Centraalbureau voor Schimmelcultures; CECT: Colección Española de Cultivos; T: Type strain
